# Generation of Phd2-haplodeficient macrophages with proresolution effects for the treatment of limb ischemia

**DOI:** 10.3389/fphar.2025.1698623

**Published:** 2025-12-19

**Authors:** Patrícia Terra Alves, Sayone Andrade de Moura, Helen Taylor, Antonella Fidanza, Cristiane Damas Gil, Leonardo Martin, Aléx Martins Nasare, André van Helvoort Lengert, Bianca Bonetto Moreno Garcia, Gabriel Cicolin Guarache, Gustavo Jose da Silva Pereira, Ivan Hong Jun Koh, Lesley M. Forrester, Sang Won Han

**Affiliations:** 1 Department of Biophysics, Escola Paulista de Medicina (EPM), Universidade Federal de São Paulo (UNIFESP), São Paulo, Brazil; 2 Department of Surgery, Escola Paulista de Medicina (EPM), Universidade Federal de São Paulo (UNIFESP), São Paulo, Brazil; 3 Centre for Regenerative Medicine, Institute for Regeneration and Repair, University of Edinburgh, Edinburgh, United Kingdom; 4 Edinburgh Medical School, Biomedical Sciences, University of Edinburgh, Edinburgh, United Kingdom; 5 Department of Morphology and Genetics, Escola Paulista de Medicina (EPM), Universidade Federal de São Paulo (UNIFESP), São Paulo, Brazil; 6 Department of Pharmaceutical Sciences, University of Antwerp, Antwerp, Belgium; 7 Infla-Med Centre of Excellence, Antwerp, Belgium; 8 Department of Pharmacology, Escola Paulista de Medicina (EPM), Universidade Federal de São Paulo (UNIFESP), São Paulo, Brazil

**Keywords:** angiogenesis, CRISPR–Cas9 gene editing, embryonic stem cell-derived macrophages, limb ischemia therapy, PHD2-haplodeficient macrophages, pro-resolving macrophages

## Abstract

**Background:**

PHD2-haplodeficient macrophages (MØs) are promising candidates for the treatment of ischemia-related conditions due to their capacity to modulate the ischemic microenvironment. However, their *in vitro* generation and therapeutic potential have not yet been established.

**Purposes:**

This study aimed to generate *Phd2* ^*+/−*^ MØs from mouse embryonic stem cells (ESCs) and to assess their functional properties *in vitro* as well as their therapeutic efficacy in a murine model of limb ischemia.

**Methods:**

*Phd2* ^*+/−*^ MØs were produced by CRISPR–Cas9-mediated disruption of one *Phd2* allele in E14IV ESCs, followed by *in vitro* differentiation. The resulting cells were characterized by flow cytometry, RT–qPCR, and functional assays to evaluate phagocytosis, angiogenic activity, and paracrine effects under normoxic and hypoxic conditions. Therapeutic efficacy was tested in a murine hindlimb ischemia model through intramuscular injection, with recovery monitored by laser Doppler perfusion imaging, muscle mass measurement, and histological analysis of regeneration and neovascularization.

**Results:**

Gene-edited E14IV ESCs yielded *Phd2* ^*+/−*^ MØs (C22-E14IV-MØs) with approximately 50% lower Phd2 expression, minimal ESC marker expression, and predominant CD206 positivity, with ∼50% of cells also expressing MHCII. In addition to generating a functional haplodeficient model, CRISPR/Cas9 editing of a non-coding intronic segment of Egln1 produced a stable ∼50% reduction in Phd2 protein levels. This quantitative decrease is compatible with a cis-acting effect on Egln1 expression, while preserving the integrity of the coding sequence. These macrophages exhibited enhanced phagocytosis, increased secretion of proangiogenic factors, and improved promotion of endothelial tube formation. *In vivo*, C22-E14IV-MØ treatment significantly enhanced blood perfusion and increased vessel formation in ischemic muscle.

**Conclusion:**

ESC-derived *Phd2* ^+/−^ MØs display robust proresolution and proangiogenic activities, promoting functional recovery in ischemic muscle. These findings support their potential as a novel cell-based therapy for ischemia-related conditions and highlight the opportunity to develop patient-specific *Phd2* ^+/−^ MØs from induced pluripotent stem cells (iPSCs) for future clinical application.

## 1 Introduction

Peripheral arterial disease (PAD) is a circulatory disorder that is caused by reduced blood flow to the limbs and primarily affects the lower limbs. PAD arises primarily as a result of chronic accumulation of lipids and fibrous tissue, which contribute to the development of atheroma plaques; these plaques obstruct the normal blood circulation over time and ultimately lead to ischemia (Fowkes et al., 2017; Morley et al., 2018). This disorder may progress to critical limb ischemia (CLI), where patients experience resting pain along with nonhealing ulcers in distal regions and gangrene (Stoyioglou and Jaff, 2004). Approximately 25%–30% of patients are not eligible for or do not respond well to the currently available treatments, surgical bypasses and endovascular procedures (Polonsky and McDermott, 2021; Stoyioglou and Jaff, 2004), so new treatments are needed.

Macrophages (MØs) play vital roles in both inflammation and repair processes within ischemic and inflamed skeletal muscle. In general, MØs are versatile cells that can adopt different functional phenotypes depending on the surrounding signals (Arnold et al., 2007; Mantovani et al., 2013; Novak et al., 2014). These cells can be broadly classified into two subtypes: M1 MØs, which are proinflammatory, and M2 MØs, which are anti-inflammatory and promote repair (Murray et al., 2014). In ischemic and inflamed skeletal muscle, a balance between M1 and M2 MØs and appropriately timed actions of these two types of MØs are critical for effective healing. If the M1 inflammatory response is too robust or prolonged, excessive tissue damage and impaired healing can occur. Conversely, if the M2 repair response is insufficient, incomplete healing and fibrosis can occur (Arnold et al., 2007; Gallo et al., 2023; Martins et al., 2020; Novak et al., 2014).

Prolyl hydroxylase domain-containing protein 2 (PHD2) is a key oxygen sensor that promotes the degradation of hypoxia-inducible factors (HIFs) under normoxic conditions (Bruick and McKnight, 2001; Ivan et al., 2001). However, under hypoxic conditions, PHD2 activity decreases, leading to the stabilization of HIFs and the subsequent activation of genes that promote angiogenesis, erythropoiesis, and vessel dilation (Semenza, 2012).

Studies conducted in mice with a heterozygous knockout of *Phd2* (*Phd2* ^*+/−*^) demonstrated that the MØs from these animals secrete factors that shape the ischemic microenvironment, resulting in increased arteriogenesis, increased recruitment and growth of muscle cells, and consequently improved recovery following an ischemic event (Hamm et al., 2013; Takeda et al., 2011). Thus, in the context of ischemia, MØs with PHD2 haploinsufficiency may contribute to PAD treatment by diminishing PHD2 activity, which would in turn facilitate the stabilization of HIFs and lead to the acquisition of an M2-like phenotype. However, patient-specific PHD2 haplodeficient MØs, which are essential for evaluating therapeutic effects in the context of human limb ischemia, are unavailable, restricting their real assessment for potential use in future treatments for limb ischemia. In theory, patient-derived PHD2 haplodeficient MØs could be generated using human induced pluripotent stem cells (iPSCs) and CRISPR‒Cas9 technology, but the potency and biosafety of these cells must first be evaluated in preclinical models.

Given this context, in this study, we generated a novel haplodeficient *Phd2* ^*+/−*^ ESC cell line by knocking out one *Egln1/Phd2* allele from the E14IV ESC line using CRISPR‒Cas9-mediated technology. The ESCs were differentiated *in vitro* to generate a pure population of *Phd2* ^*+/−*^ MØs, and the phenotype and function of these cells were compared to those of MØs generated from ESCs without genetic modifications. The therapeutic potential of *Phd2* ^*+/−*^ MØs was assessed in a murine model of hind limb ischemia.

## 2 Materials and methods

### 2.1 Vector construction

**FIGURE 1:**
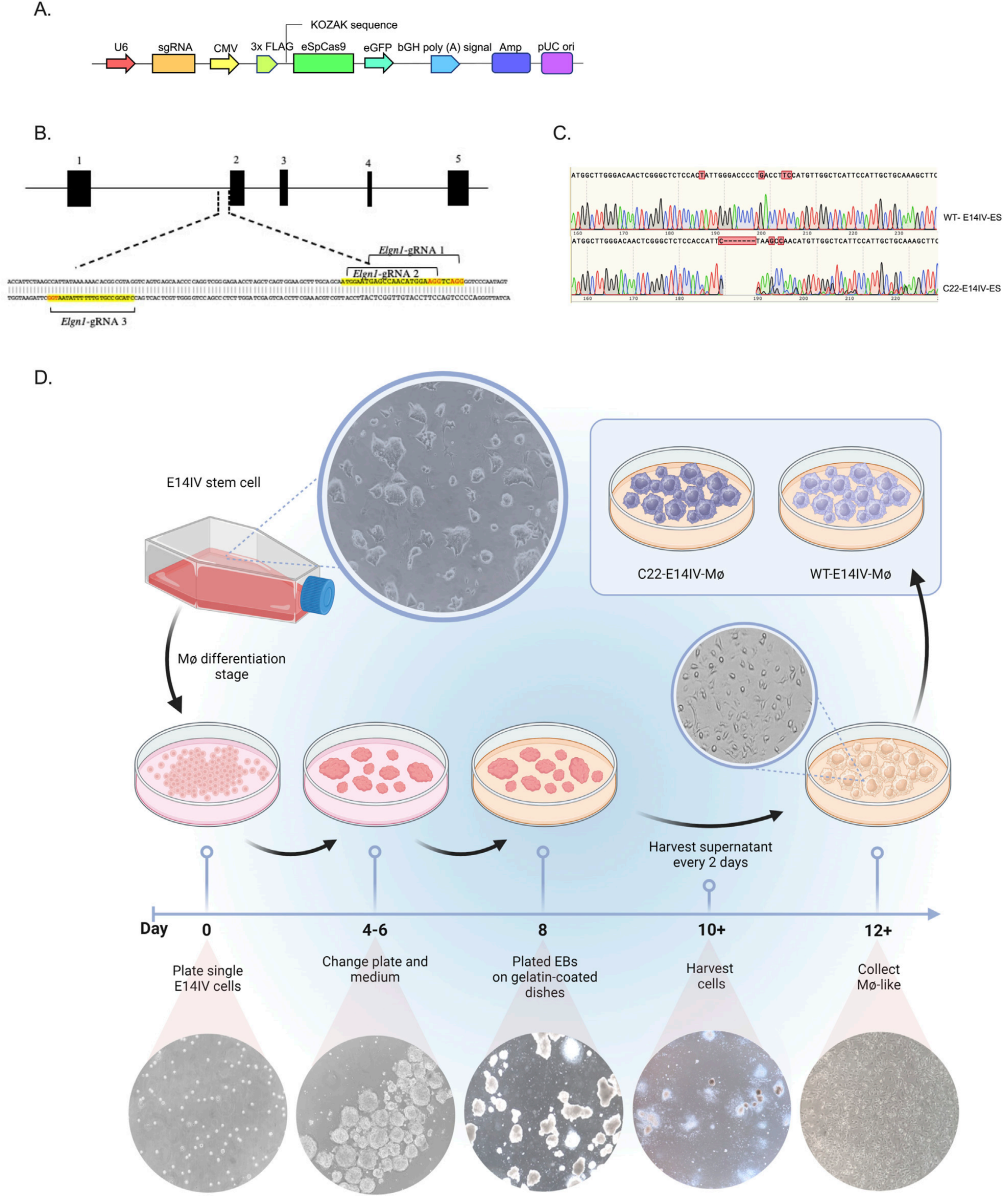
Genetic engineering of E14IV cells with CRISPR‒Cas9 to produce *Phd2* ^*+/−*^ MØs **(A)** Linear map of the plasmid (pCAG-eCas9-GFP-U6-gRNA) used to edit the E14IV-ES cell genome with CRISPR‒Cas9. **(B)** Schematic diagram of the *Phd2* gene, with exons depicted as black boxes. The gRNAs and protospacer-adjacent motif (PAM) sequences are highlighted in yellow and red, respectively. The sequence is presented in the inverted orientation with respect to the gene. **(C)** Sequencing of DNA from the targeted site in wild-type (WT-E14IV-ES) and clone 22 (C22-E14IV-ES) cells. **(D)** Schematic diagram of the protocol used to produce MØs from E14IV-ES cells. On Day 0, E14IV-ES cells (6 x10^5^) were seeded in a 95-mm Petri dish and cultured in suspension for 8 days to form embryoid bodies (EBs). On Days 4 and 6, the EBs were transferred to a new Petri dish containing fresh medium to support their growth and maturation. On Day 8, the EBs were harvested and plated in gelatin-coated Petri dishes. Starting on Day 10, the supernatant containing the MØ progenitor cells was collected every 2 days and plated onto 95-mm Petri dishes. The MØ progenitor cells adhered to the dish and differentiated into MØ-like cells, which could then be cultured for 7 days (Day 12+). Microscopy images (10×) show the cell morphology at various stages of differentiation. High-resolution representative images of undifferentiated E14IV-ES cells and differentiated macrophages are provided in Supplementary Figure S2.

The Phd2-gRNAs were designed using Sanger CRISPR software (https://www.sanger.ac.uk/htgt/wge/find_crisprs), and the associated details can be found in Supplementary Table S1. Synthetic single-stranded DNA oligonucleotides (ssODNs) corresponding to the designed crRNA sequences were purchased from Integrated DNA Technologies (United States). Each oligonucleotide contained short additional sequences at both the 5′ and 3′ ends to facilitate ligation into the pCAG-eCas9-GFP-U6-gRNA vector (Addgene, Watertown, MA, United States), which was linearized with BbsI. The complementary oligonucleotides were then annealed and ligated following standard molecular cloning procedures. The resulting ligation product was used to transform *Escherichia coli* Top10. Analysis of the transformed clones was carried out via Sanger DNA sequencing. The final product was referred to as pCAG-eCas9-GFP-U6-gRNA (Figure 1A).

### 2.2 Mouse embryonic stem cell maintenance and MØ differentiation

E14IV-ES cells were maintained as previously described (Jackson et al., 2010). The maintenance medium consisted of Glasgow minimum essential medium (Sigma, St. Louis, MO, United States, #G5154) supplemented with 10% embryonic stem cell fetal bovine serum (FBS) (Thermo Fisher Scientific, Waltham, MA, United States, #10439024), 2 mM sodium pyruvate (Thermo Fisher Scientific, # 11360039), 4 mM L-glutamine (Sigma, #G7513), 1% nonessential amino acids (Thermo Fisher Scientific, #111400), 0.1 mM β-mercaptoethanol (Sigma, #31350-010), and 100 U/mL leukemia inhibitory factor (LIF) (Sigma, # L5283-10) (Haideri et al., 2017; Jackson et al., 2010). For MØ production, the same medium was used, excluding LIF, with a switch to standard FBS (Thermo Fischer Scientific, #A5256701), plus 15% L929-conditioned medium (CM) and 1 ng/mL recombinant IL-3 (Stem Cell Technologies, Vancouver, Canada, #780421) (Figure 1D).

### 2.3 Preparation of bone marrow-derived macrophages (BM-MØs)

BM-MØs were obtained from 8- to 10-week-old 129SV/WT mice following a previously described method (Haideri et al., 2017). Briefly, the mice were euthanized, and their femurs, tibias, and humeri were collected. The bones were flushed to extrude the bone marrow, and the resulting cell suspensions were cultured on 10-cm diameter polystyrene plates for 7 days in RPMI-1640 medium (Thermo Fischer Scientific, # 11875085) containing 10% heat-inactivated FBS (Thermo Fischer Scientific, #A5256701), 30% L929 CM, and 1% penicillin/streptomycin solution (Thermo Fischer Scientific, # 15140122) (with fresh medium added on Day 4). BM-MØs were harvested from the plates by vigorous pipetting of ice-cold PBS and used in subsequent experiments.

### 2.4 Culture of the C2C12 cell line

C2C12 myoblasts derived from mouse skeletal muscle were cultured following the recommendations from the American Type Culture Collection (ATCC, Manassas, VA, United States). The cells were cultured in Dulbecco’s modified Eagle medium (DMEM, Thermo Fischer Scientific, # 11965092) supplemented with 10% FBS and 2 mM L-glutamine and maintained in a humidified atmosphere at 37 °C with 5% CO_2_.

### 2.5 Genetic engineering of E14IV cells with CRISPR‒Cas9 to generate the *Phd2* ^+/−^ genotype

The pCAG-eCas9-GFP-U6-gRNA + PHD2-gRNA plasmid (Addgene #79145; Figure 1A) was used for transfection of E14IV-ES cells (1 × 10^6^ cells per well in a 6-well plate) with 5 μg of plasmid DNA using the Xfect Transfection Kit (TaKaRa, Shiga, Japan, # 631317), following the manufacturer’s protocol. Twenty-four hours after transfection, the cells were sorted to obtain single cells and collected in 96-well plates using an LSRFortessa machine (BD Bioscience, Franklin Lakes, NJ, United States). When the cells reached confluence, the clones were transferred to 24-well plates and then to 6-well plates. The clones were subsequently screened on the basis of Phd2 protein expression and subsequently frozen.

To confirm the presence of the expected genomic alteration, genomic DNA was extracted using the MasterPure™ Complete DNA and RNA Purification Kit (Epicenter, Madison, WI, United States, #MC85200) following the manufacturer’s instructions. PCR amplification was performed using the Prime Star Max DNA Polymerase Kit (TaKaRa, Shiga, Japan, #R045B), with forward (5′-TTT​GTA​GTT​GGC​CGT​TCA​GAG​TGG​GG-3′) and reverse (5′-ATA​GGT​GCG​TAC​TGC​ACA​AGG​CAT​GC-3′) oligonucleotides to generate a fragment of approximately 800 bp. The amplicon was purified using a QIAquick PCR Purification Kit (Qiagen, Sao Paulo, Brazil, #28104) and sequenced by the Sanger sequencing method. The electropherograms obtained via Sanger sequencing of amplicons from both the plasmid and the empty vector were subjected to indel analysis with the TIDE (https://tide.deskgen.com.br) and Synthego ICE CRISPR analysis tools (https://www.synthego.com/products/bioinformatics/crisprr-analysis) (Brinkman et al., 2014; Roginsky, 2018).

### 2.6 Phd2 protein expression analysis

The cells from the harvested clones were homogenized and digested using RIPA buffer (Thermo Fisher Scientific) with a protease inhibitor cocktail (Sigma) to prepare extracts. The protein concentration was determined using the Pierce Rapid Gold BCA Protein Assay Kit (Thermo Fisher Scientific). NuPAGE LDS sample buffer (Thermo Fisher Scientific) with 1% (v/v) 2-mercaptoethanol diluted (1:4) in RIPA buffer was used to prepare a protein concentration of 100 μg/mL. The protein was denatured by heating (70 °C; 10 min), and 20 μg of protein was loaded onto a NovexTM WedgeWell™ 4%-20% Tris-glycine gel (Thermo Fisher Scientific) and electrophoresed (225 V; 30 min). The proteins were then transferred to 0.45-µm nitrocellulose membranes (GE Healthcare, Sao Paulo, Brazil) at 100 V (90 min) on ice in transfer buffer composed of 48 mM Tris-HCl, 39 mM glycine, and 20% methanol. After transfer, the membranes were blocked with 5% blocking-grade blocker (Bio-Rad, United States) in TBS-T (20 mM Tris-HCl, pH 7.5, 150 mM NaCl, and 0.1% (v/v) Tween 20) at room temperature (RT) for 1 h. The membranes were then washed once with TBS-T and incubated overnight (4 °C) with primary antibodies diluted in 5% bovine serum albumin (BSA) in TBS-T. The membranes were again washed 3 times with TBS-T, incubated with the secondary antibody diluted in TBS-T at room temperature with shaking (1 h) and then washed three times with TBS-T. Proteins were visualized using the Bio-Rad ChemiDoc System (Bio-Rad, Sao Paulo, Brazil), images were evaluated, and intensity was quantified using Bio-Rad ImageLab software. The blot was stripped and reprobed with antibodies specific for Gapdh, and the ratio of optical density of Phd2 to that of Gapdh was calculated. The antibodies used in this experiment were anti-Phd2/Egln1 (rabbit, 1:1,500, Cell Signaling, Danvers, MA, United States), anti-Hrp (horseradish peroxidase; goat, 1:1,000 Abcam, Cambridge, United Kingdom), and anti-Gapdh (goat, 1:5,000, R&D, Minneapolis, MN, United States).

### 2.7 MØ immunophenotyping

The MØs were harvested and blocked with Fc blocking reagent (BD Bioscience) for 10 min on ice. A total of 1 × 10^5^ cells were washed and stained with the appropriate antibodies (20 min; room temperature). Dead cells were gated out using DAPI. The anti-mouse antibodies used were as follows: anti-F4/80 APC (1:100; BioLegend, United States), anti-CD11b Alexa Fluor 488 (1:100; BioLegend, United States), anti-MHCII APC/Cy7 (1:100; BioLegend, United States), anti-CD206 PE (1:300; BioLegend, United States), and anti-SSEA1 Alexa Fluor 647 (1:1,000; BioLegend, United States). The MØs were then washed in PBS with 1% BSA and 5 mM EDTA and kept on ice for less than 1 h before analysis. Flow cytometry analysis was performed using an LSRFortessa Analyzer (BD Bioscience) and FlowJo software (v. 10.4).

### 2.8 MØ gene expression profiling

For RT‒qPCR, total RNA was extracted with the RNeasy Mini Kit (Qiagen), and cDNA was prepared with 500 ng of total RNA using the high-capacity cDNA synthesis kit (Thermo Fisher Scientific). Two nanograms of cDNA was amplified per reaction, and each reaction was performed in triplicate using the LightCycler 384 system with SYBR Green Master Mix I (Roche, Swiss). The *Gapdh* gene was used as a reference. The primer sequences and efficiency values are listed in Supplementary Table S2 in the online Data Supplement.

### 2.9 Phagocytosis assay

The phagocytosis assay was performed according to Haideri et al. (Haideri et al., 2017). Briefly, E14IV-MØs (1 × 10^5^) were plated in 96-well plates (Cell Carrier, Perkin Elmer, Sao Paulo, Brazil). Next, the cells were washed with PBS, and the nuclei (NucBlue Live ReadyProbes Reagent, Thermo Fisher Scientific) and membranes were stained (CellMask™ deep red plasma membrane stain; Thermo Fisher Scientific) according to the manufacturer’s instructions. The pHrodo™ Green Zymosan A BioParticles Conjugate (Thermo Fisher Scientific) was used to evaluate the phagocytic capacity of E14IV-MØs with the Operetta High-Content Imaging System (Perkin Elmer). Analysis was performed using Harmony High-Content Imaging and Analysis software (Perkin Elmer).

### 2.10 Phd2 expression under normoxic and hypoxic conditions

To analyze the influence of hypoxia on Phd2 protein expression, E14IV-ES cells or E14IV-MØs (1.5 × 10^6^ cells) were seeded on a 6-well plate overnight in a CO_2_ chamber (5% CO_2_; 37 °C). For incubation under normoxic conditions, the plate was maintained in the same CO_2_ chamber for 2 h. For incubation under hypoxic conditions (37 °C, 1% O_2_, 5% CO_2_, and 94% N_2_), the plate was transferred to a multigas incubator (*in vitro* Cell ES NU-5831E, Nuaire, Plymouth, MN, United States) and maintained for 2 h Phd2 protein expression in the samples was assessed according to the protocol described above.

### 2.11 Proteomic analysis of angiogenesis-related genes

A Proteome Profiler Mouse Angiogenesis Antibody Array (R&D Systems) was used according to the manufacturer’s instructions. Briefly, nitrocellulose membranes spotted with antibodies specific for different angiogenesis-related proteins were first incubated in blocking buffer (1 h). CMs prepared by incubation of 1 × 10^6^ MØs (BM-MØs, WT-E14-MØs and C22-E14IV-MØs) in the appropriate media overnight were collected. Five hundred microliters from each sample and PBS were mixed, and a cocktail of biotin-labeled antibodies specific for different angiogenesis-related proteins was added. This solution was placed onto the membranes and incubated overnight (4 °C). The membranes were then washed and incubated with HRP-conjugated streptavidin (1 h; RT), and the signals were quantified according to the manufacturer’s protocol using Image Lab software (Bio-Rad).

### 2.12 Tube formation assay

The ability of E14IV-MØs to promote endothelial cell tube formation was assessed using Matrigel. Human umbilical vein endothelial cells (HUVECs) and MØs, either alone (11,000 cells each) or in combination (10,000 HUVECs and 1,000 MØs), were seeded on Matrigel (Corning, Sao Paulo, Brazil) in Ibidi plates (Ibidi, Gräfelfing, Germany) (DeCicco-Skinner et al., 2014). After 2 h, as tube formation began, images were captured with a Zeiss Observer Z1 and further analyzed using AxioVision microscopy (20X) software. Tube formation was evaluated using the Angiogenesis Analyzer tool in ImageJ software. BM-MØs were not included in the tube formation assay because their angiogenic activity was already confirmed by the angiogenic proteome assay (Figure 3A). This approach allowed a focused comparison between WT- and C22-E14IV-MØs and the baseline condition represented by HUVECs cultured alone.

### 2.13 Paracrine effects of MØs on C2C12 cell migration and proliferation

The paracrine effect of MØs was carried out using macrophage conditioned medium (MØ-CM). To prepare MØ-CM, MØs (1.5 × 10^6^) were seeded on a 6-well plate overnight in a CO_2_ chamber (5% CO_2_; 37 °C). The cells were washed two times with PBS, and the medium was replaced with fresh medium. For collection of MØ-CM under normoxic conditions, the plate was maintained in the same CO_2_ chamber (2 h). For collection of MØ-CM under hypoxic conditions (1% O_2_, 5% CO_2_, and 94% N_2_), the plate was transferred to a hypoxic incubator chamber (2 h) (Stem Cell). Hypoxic conditions were generated according to the manufacturer’s recommendations. After MØ-CM was produced, the medium were centrifuged (Eppendorf, Sao Paulo, Brazil) at 300 × g (5 min), filtered through a 0.22 µm filter (Millipore, Sao Paulo, Brazil), and then stored at −70 °C until use.

For the wound healing and migration assay, CMs from BM-MØs, WT-E14IV-MØs, and C22-E14IV-MØs were prepared under normoxic and hypoxic conditions. C2C12 cells cultured (90%–100% confluence) in 24-well plates were mechanically scratched with a sterile 200 µL pipet tip. The wells were washed twice with PBS, and new medium containing 10 μg/mL mitomycin C (Sigma) and 40% MØ-CM was added. In the control group, the cells were cultured without MØ-CM. The cells were maintained for 12 h (37 °C; 5% CO_2_) on an Axio Observer Z1 incubation microscope (Zeiss, Oberkochen, Germany), and images were acquired with a 10× objective during this time. The wound closure area was evaluated using TScrash analysis software and plotted for statistical analysis (Geback et al., 2009).

For the cell proliferation assay, the growth of C2C12 cell was monitored with the CellTrace CFSE Cell Proliferation Kit (Thermo Fisher Scientific) according to the manufacturer’s instructions. Briefly, the cells were stained with 5 μM carboxyfluorescein diacetate succinimidyl ester (CFSE) in PBS +0.1% BSA (37 °C; 10 min). The stained cells were plated on 6-well plates at a density of 5 × 10^5^ cells/well in 2 mL of DMEM with 10% FBS and maintained overnight in a CO_2_ chamber (5% CO_2_; 37 °C). Then, the cells were washed with PBS to remove nonadherent cells, and fresh medium containing 40% CM was added. In the control group, the cells were cultured without MØ-CM. After 24 h, the cells were collected, and CFSE decay was analyzed with a FACSCanto II flow cytometer (BD Biosciences) to estimate the proliferation rate. The acquired data were analyzed using FlowJo software (BD Biosciences).

### 2.14 Cell therapy for the treatment of hindlimb ischemia (HLI)

HLI was induced in 12- to 14-week-old male 129SV/WT mice following previously described procedures, with some modifications (Martins et al., 2014; Padgett et al., 2016; Sacramento et al., 2009). Briefly, after anesthesia was induced with 4% isoflurane (2-chloro-2-(difluoromethoxy)-1,1,1-trifluoroethane) and subsequently maintained with 1%–2% isoflurane in oxygen. HLI was then induced with two ligatures on the left femoral artery in the region proximal to the bifurcation of the femoral artery into the popliteal artery and saphenous artery, followed by transection between the ligatures (1 mm apart). On Day 3 post-ischemia, MØs suspended in saline solution (60 μL; 5 × 10^5^ cells) were injected into the gastrocnemius muscle. All ischemic mice received oral tramadol treatments (20 μL; 24 mg/mL) in the morning and in the evening to help manage pain during the first 4 days after surgery. In this study, the following groups (5 mice per group) were included: nonischemic (NI), sham (S), ischemic (I), ischemic treated with BM-MØs (I + BM-MØ), ischemic treated with WT-E14IV-MØs (I + WT-E14IV-MØ), and ischemic treated with C22-E14IV-MØs (I + C22-E14IV-MØ). For animal identification, mice were marked by ear punching using a numbered ear code system, following institutional guidelines and approved ethical protocols.

To quantify superficial blood flow, the ischemic and contralateral hindlimbs were monitored using a laser Doppler imaging (LDI) system (moorLDI2-IR; Moor Instruments, Axminster, United Kingdom) (Gohin et al., 2020). The ratio of blood flow between the ischemic and contralateral hindlimbs was calculated and used as a perfusion index. These measurements were performed on Days 1, 7, 14, and 21 after HLI. On Day 21, the animals were euthanized, and their gastrocnemius muscles were collected for mass quantification and histological evaluation. For euthanasia, mice received an intraperitoneal injection of ketamine (300 mg/kg) and xylazine (30 mg/kg) diluted in saline. Death was confirmed by the absence of reflexes, after which the animals were disposed of in accordance with institutional guidelines.

For histological evaluation, the gastrocnemius muscles were collected, fixed in 4% paraformaldehyde (24 h), and subsequently embedded in optimal cutting temperature solution (OCT) (EasyPath, Brazil). We prepared 5 μm cryosections and stained them with hematoxylin-eosin (H&E) (Eosin Yellowish 0.5%; Harry’s hematoxylin; EasyPath, Indaiatuba, Brazil) for histomorphometry and Masson’s trichrome (EasyPath) for collagen distribution analysis. Immunohistochemical staining was performed to examine the capillaries and arterioles using antibodies specific for CD31 (1:50, Abcam) and alpha-smooth muscle actin (*α*-SMA) (1:50, Dako, Glostrup, Denmark), respectively, following a previously described protocol (Martins et al., 2020). The quantification of fiber muscles and blood vessels (arterioles and capillaries) was performed in 3 fields/section using a 20× objective on an Axio Scope A1 Zeiss microscope (Carl Zeiss, Jena, Germany), and the area was determined with AxioVision software (Carl Zeiss). We categorized muscle fibers on the basis of three morphological criteria: cells with peripheral nuclei (normal), cells with central nuclei (immature), and damaged cells. All data are presented as the mean ± standard deviation (SD) of the number of cells or blood vessels/mm^2^.

### 2.15 Statistical analysis

All statistical analyses were performed using Prism 7 software (GraphPad; San Jolla, CA, United States). The results are expressed as the means ± SDs. For comparisons among multiple groups, one-way ANOVA followed by Tukey’s multiple comparison test was applied.

For analyses involving two independent variables, a two-way ANOVA was used, followed by Tukey’s *post hoc* test when appropriate. Differences were considered statistically significant at *p* < 0.05. The statistical methods used are detailed in the corresponding figure legends.

## 3 Results

### 3.1 Genetic engineering of E14IV cells with CRISPR-Cas9 to produce *Phd2* ^*+/−*^ MØs

Three gRNAs (Supplementary Table S1), which are complementary to sequences near exon 2 of the *Phd2* gene, were designed to induce indels, enabling the generation of selectable clones haplodeficient for Phd2 (Figure 1). The genome editing results indicated that the efficiency of *Phd2*-gRNA1 was 25.4% according to TIDE analysis and that there were19.4% indels and 17% knockouts with this gRNA according to Synthego ICE analysis. The other gRNAs presented lower efficiency and were therefore discarded. Twenty-four hours after transfection with eCAS9-GFP-PHD2-gRNA1, ESCs were sorted as single cells, and 180 clones were isolated. This population exhibited a total mutation efficiency of 45.7% according to TIDE analysis, with 69% indels and 50% knockouts according to Synthego ICE analysis. The Phd2 protein levels of each clone were assessed by Western blotting and compared with those of the control wild-type ESC line (WT-E14IV-ES) (Supplementary Figure S1). One clone, referred to as C22-E14IV-ES, presented significantly reduced Phd2 protein levels (43% of control ESC levels). TIDE analysis revealed that this clone harbored a seven-nucleotide deletion in one allele, which caused a frameshift mutation, resulting in an out-of-frame transcript and a predicted absence of functional Phd2 protein from the disrupted allele (Figure 1C). The C22-E14IV-ES clone was selected for further differentiation studies and phenotypic assessment of MØs (Figure 1D).

### 3.2 MØ characterization

**FIGURE 2:**
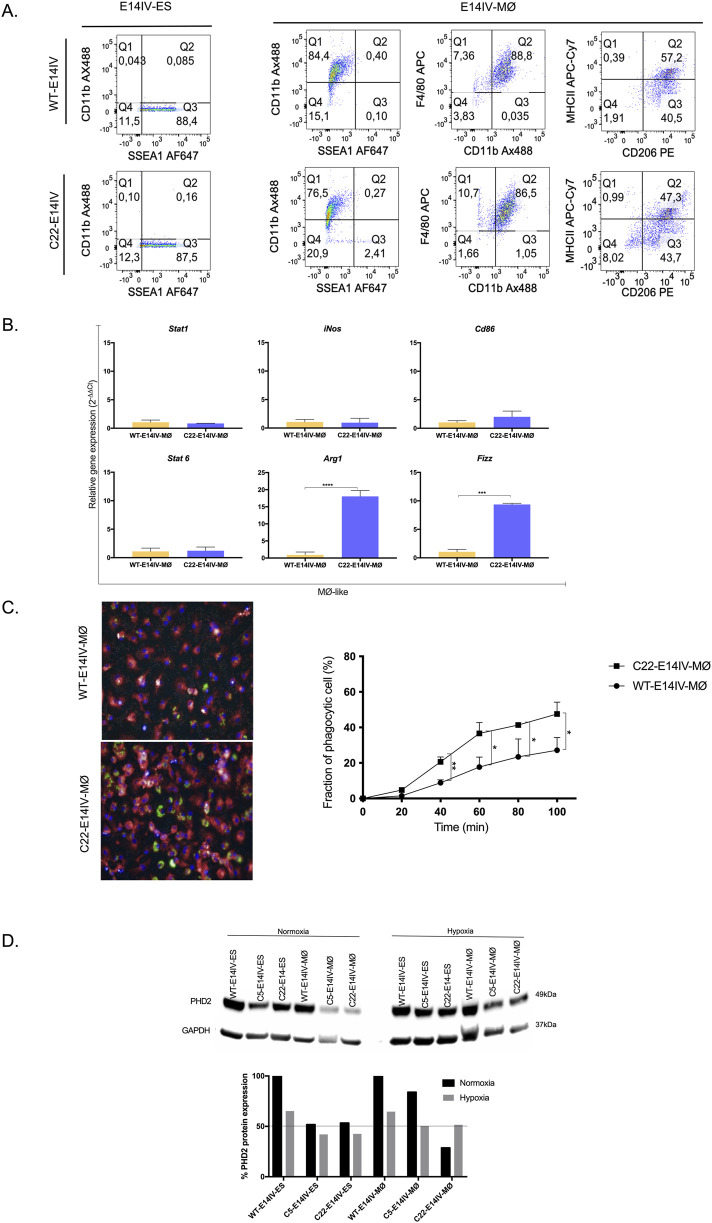
Characterization of MØs derived from E14IV-ES cells **(A)** Flow cytometry-based immunophenotyping of E14IV-ES cells and E14IV-MØs using SSEA1 (pluripotent stem cell marker) and F4/80 and CD11b (MØ markers). The F4/80+ CD11b+ double-positive population was further analyzed for the expression of MHCII (an M1-like marker) and CD206 (an M2-like marker). **(B)** Gene expression analysis of key MØ markers in E14IV-MØs by RT‒qPCR: *Stat1, iNos,* and *Cd86* are phenotypic markers for M1-like MØs; *Stat6* and *Arg1* are phenotypic markers for M2-like MØs; and *Fizz* is a MØ activation marker. **(C)** Phagocytosis assay of WT-E14IV-MØs and C22-E14IV-MØs. Timing started after pHrodo beads were added to the cells, and the green fluorescence of the beads was quantified with Harmony image analysis software. Representative time-lapse images from this assay at 0–100 min are provided in Supplementary Figure S3. **(D)** Quantification of Phd2 protein expression in E14IV-ES cells, E14IV-MØs and C22-E14IV-MØ cultured for 2 h under normoxic or hypoxic conditions. The expression level in WT-E14IV-ES cells or WT-E14IV-MØs under normoxic conditions was considered 100% for relative quantification of Phd2 protein expression. One-way ANOVA with Tukey’s *post hoc* test was performed for multiple comparisons (*p < 0.05; **p < 0.01).

WT-E14IV-ES and C22-E14IV-ES cells were differentiated into WT-E14IV-MØs and C22-E14IV-MØs, respectively, and their phenotypes and functions were compared. Flow cytometry analysis revealed that WT-E14IV-MØs and C22-E14IV-MØs were negative for the stem cell marker SSEA1 (less than 3%), and approximately 90% of the cells were double-positive for the F4/80 and CD11b MØ markers (Figure 2A). The MØ marker-positive populations were further analyzed for MHCII and CD206 expression, representing M1 and M2 MØs, respectively, by flow cytometry. Two major MØ populations were identified on the basis of their expression profiles: one with an M2-like profile (F4/80^+^CD11b^+^MHCII^−^CD206^+^) and another with both M1-like and M2-like profiles (F4/80^+^CD11b^+^MHCII^+^CD206^+^) (Figure 2A).

The gene expression of key MØ markers in WT-E14IV-MØs and C22-E14IV-MØs was assessed by RT‒qPCR. No significant differences in the expression of genes associated with M1-MØs (*Stat1*, *iNos*, and *Cd86*) were detected. However, compared with WT-E14IV-MØs, C22-E14IV-MØs presented significantly greater expression levels of the M2-associated genes *Arg1* and *Fizz*, with 18-fold and 9-fold increases, respectively (Figure 2B).

As phagocytosis is one of the main functions of MØs, this process was evaluated in WT-E14IV-MØs and C22-E14IV-MØs. After 40 min, phagocytic activity was evident in both cell lines, with C22-E14IV-MØs displaying nearly twice the phagocytic activity of E14IV-MØs. Beyond this time point, phagocytic activity continued to increase until 100 min, with a statistically significant difference between the two populations (Figure 2C).

Phd2 protein expression in WT-E14IV and C22-E14IV ESCs, as well as their respective MØs, was compared under hypoxic and normoxic conditions (Figure 2D). As expected, Phd2 protein expression in WT-E14IV-ES cells and WT-E14IV-MØs under hypoxic conditions decreased to approximately 63% of the levels observed under normoxic conditions. In contrast, Phd2 protein expression in C22-E14IV-ES cells and C22-E14IV-MØs under normoxic conditions was approximately half that in the corresponding WT-E14IV cells, reflecting haploinsufficiency due to the disruption of one *Phd2* allele. Notably, culturing C22-E14IV cells under hypoxic conditions for 2 h did not further reduce Phd2 protein levels.

### 3.3 MØ angiogenic activity

**FIGURE 3:**
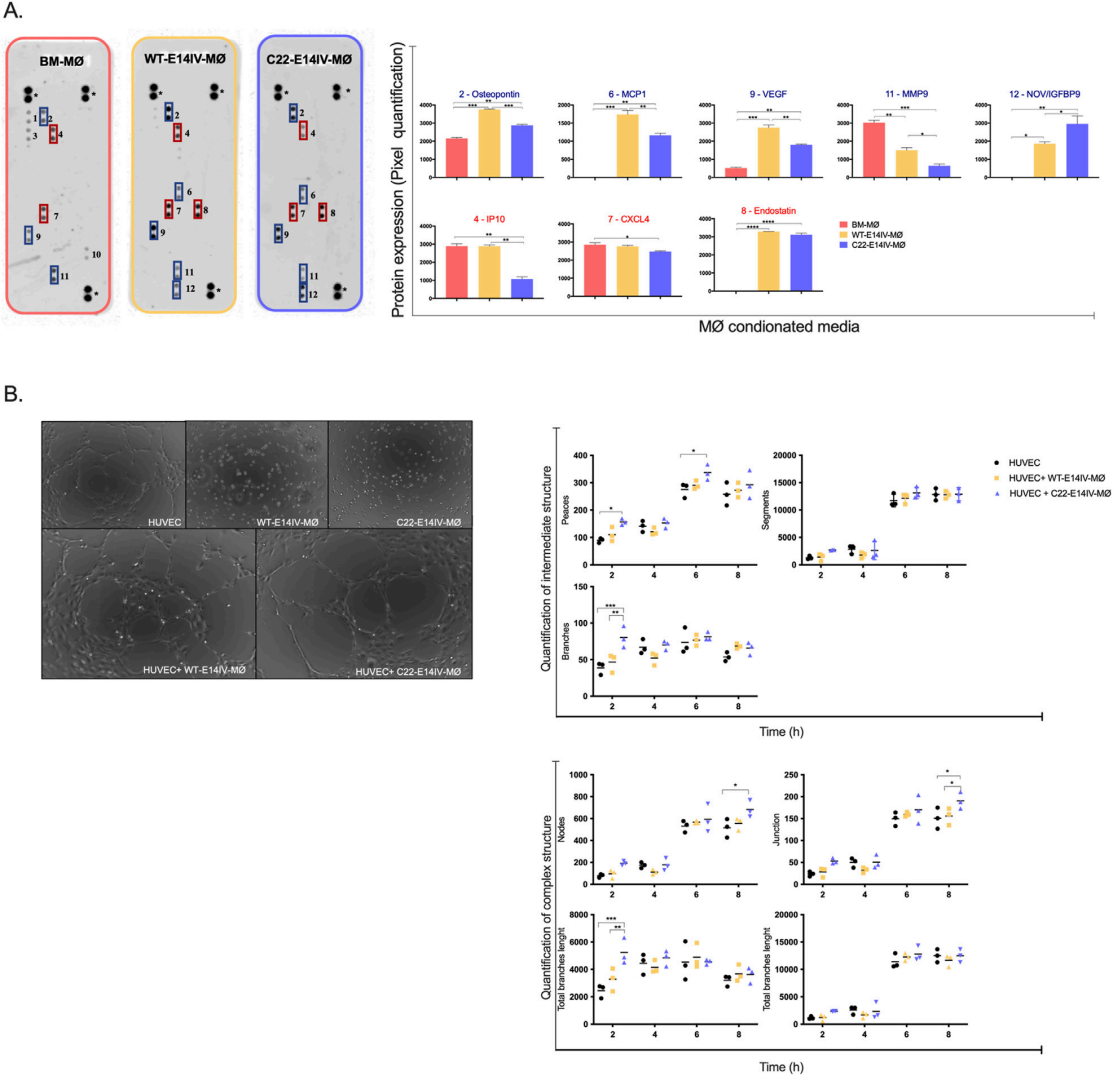
Angiogenesis induction capacity of MØs **(A)** Representative images of angiogenic factor protein arrays performed using conditioned media from BM-MØs, WT-E14IV-MØs, and C22-E14IV-MØs. The proteins in the membranes were as follows: * Positive control; 1- Cxcl12; 2- Osteopontin; 3- Serpin E1; 4- Ip-10 (Cxcl10); 5- KC (Cxcl1); 6- Mcp1 (Ccl12/JE); 7- Cxcl4; 8-Endostatin/Collagen XVIII; 9- Vegf; 10- Cxcl16; 11- Mmp9; and 12- Nov (Ccn3, Igfbp-9). The dots were quantified by densitometry, and the results were plotted as graphs. **(B)** Representative images of tube formation resulting from the interaction between HUVECs and WT-E14IV-MØs or C22-E14IV-MØs. The images were analyzed to quantify intermediate structures (branches and segments) and complex structures (nodes, junctions, total branch length, and total segment length). Two-way ANOVA with Tukey *post hoc* analysis was performed for multiple comparisons (*p < 0.05; **p < 0.01; ***p < 0.001).

The angiogenic activity of MØs was evaluated using both an angiogenic proteome assay and a tube formation assay. The proteome array identified 12 angiogenesis-related proteins out of 52 analyzed, with 8 proteins found to be present specifically in the E14V-MØ groups (Figure 3A). The proteome findings revealed a marked increase in the levels of several proangiogenic factors in the supernatants of WT-E14IV-MØs and C22-E14IV-MØs compared with those of BM-MØs. These proangiogenic factors included Osteopontin (BM-MØs: 2,153 ± 57; WT-E14IV-MØs: 3,756 ± 20; C22-E14IV-MØs: 2,871 ± 61), Monocyte chemotactic protein-1 (MCP1) (BM-MØs: not detected; WT-E14IV-MØs: 1741 ± 106; C22-E14IV-MØs: 1,161 ± 60), Vascular Endothelial Growth Factor (VEGF) (BM-MØs: 520 ± 46; WT-E14IV-MØs: 2,752 ± 144; C22-E14IV-MØs: 1806 ± 28), and Nephroblastoma Overexpressed Protein (NOV/IGFB9) (BM-MØs: not detected; WT-E14IV-MØs: 1857 ± 104; C22-E14IV-MØs: 2,956 ± 500).

In contrast, Matrix Metalloproteinase 9 (MMP9) levels were significantly higher in the supernatant from BM-MØs than in those from both E14IV-MØ populations (BM-MØs: 3,028 ± 128; WT-E14IV-MØs: 1,502 ± 138; C22-E14IV-MØs: 639 ± 100). Antiangiogenic factors, including IFN-gamma-inducible protein 10 (Ip-10) and CXC chemokine ligand 4 (CXCL14), were secreted in comparable amounts by both WT-E14IV-MØs (2,894 ± 65 and 2,766 ± 52, respectively) and BM-MØs (2,899 ± 129 and 2,850 ± 113, respectively). However, C22-E14IV-MØs produced significantly lower levels of these factors (1,069 ± 130 and 2,475 ± 32, respectively). Notably, endostatin, an antiangiogenic protein, was not detected in BM-MØs but was present in high amounts in E14IV-MØs (WT-E14IV-MØs: 3,285 ± 6; C22-E14IV-MØs: 3,115 ± 90). Therefore, all MØ populations exhibited proangiogenic profiles, each characterized by a distinct set of features.

To evaluate angiogenic activity during endothelial tube formation, HUVECs were cocultured with C22-E14IV-MØs or WT-E14IV-MØs or maintained alone as controls. The inclusion of HUVECs cultured alone served to determine the intrinsic capacity of E14IV-derived macrophages to promote angiogenic tube formation. Although bone marrow–derived macrophages (BM-MØs) were not included in this assay, their angiogenic potential was already demonstrated in the proteome analysis (Figure 3A), where BM-MØs secreted several pro-angiogenic proteins such as osteopontin, MMP9, and VEGF. Therefore, the tube formation assay was designed to compare the specific angiogenic potency of E14IV-derived macrophages (WT and C22) relative to HUVECs alone, rather than to reproduce the BM-MØ effect, which had already been characterized in the proteomic profile.

Images of the HUVECs were analyzed to quantify the following parameters: the numbers of nodes and junctions and the total length of branches and segments. Each parameter was characterized as follows: number of nodes: the number of points where multiple branches meet, indicative of network complexity; number of branches: the total number of connections between nodes, reflecting the degree of vascular connectivity; branch length: the total length of individual tube connections (branches) between two junctions or nodes; segment length: the lengths of the linear parts of tubes between any two connection points (e.g., nodes or endpoints), with it possible for a segment to include branches or be part of a branch; number of segments: the number of continuous linear sections of tubes between two nodes or endpoints; and number of junctions: the number of points where three or more branches converge, reflecting the maturity of the network.

Following this quantification, the parameters were classified as intermediate or complex structures. Intermediate structures represent the early stages of the angiogenic process, typically consisting of simple, linear tubes with minimal branching. For these structures, the number of branches and segments was evaluated. Complex structures represent more advanced stages of network formation, characterized by intricate branching and interconnected loops. For these structures, the numbers of nodes and junctions and the total lengths of branches and segments were evaluated.

At early time points (2 h), C22-E14IV-MØs presented a significantly greater number of branches than did WT-E14IV-MØs and HUVECs alone (p < 0.001) (Figure 3B). This difference diminished over time, and by 4–8 h, all the conditions resulted in a similar number of branches. Segment counts were not significantly different among the three groups across all time points. The parameters used to evaluate intermediate structures indicated that while C22-E14IV-MØs exhibit enhanced branch formation, this does not translate into a significant difference in total segment numbers, indicating that segments may not be a sensitive measure of intermediate structure differences in this context.

C22-E14IV-MØs consistently showed a slightly greater number of nodes than the other conditions did, particularly at later time points (6–8 h), but this difference was not statistically significant. Compared with WT-E14IV-MØs, C22-E14IV-MØs presented a significantly greater number of junctions at 6 h (p < 0.05). In terms of total branch length at 2 h, C22-E14IV-MØs significantly outperformed both WT-E14IV-MØs and HUVECs alone (p < 0.001). The total segment length was not significantly different between the groups at any time point. These results indicate that, compared with WT-E14IV-MØs and HUVECs alone, C22-E14IV-MØs exhibit superior angiogenic activity. The key parameters highlighting this effect include the numbers of branches and junctions and the total branch length, which significantly differed at specific time points. By 8 h, the observed differences between conditions diminished, suggesting that other factors might limit further network progression.

### 3.4 Paracrine effects of MØs on myoblast migration and proliferation

**FIGURE 4:**
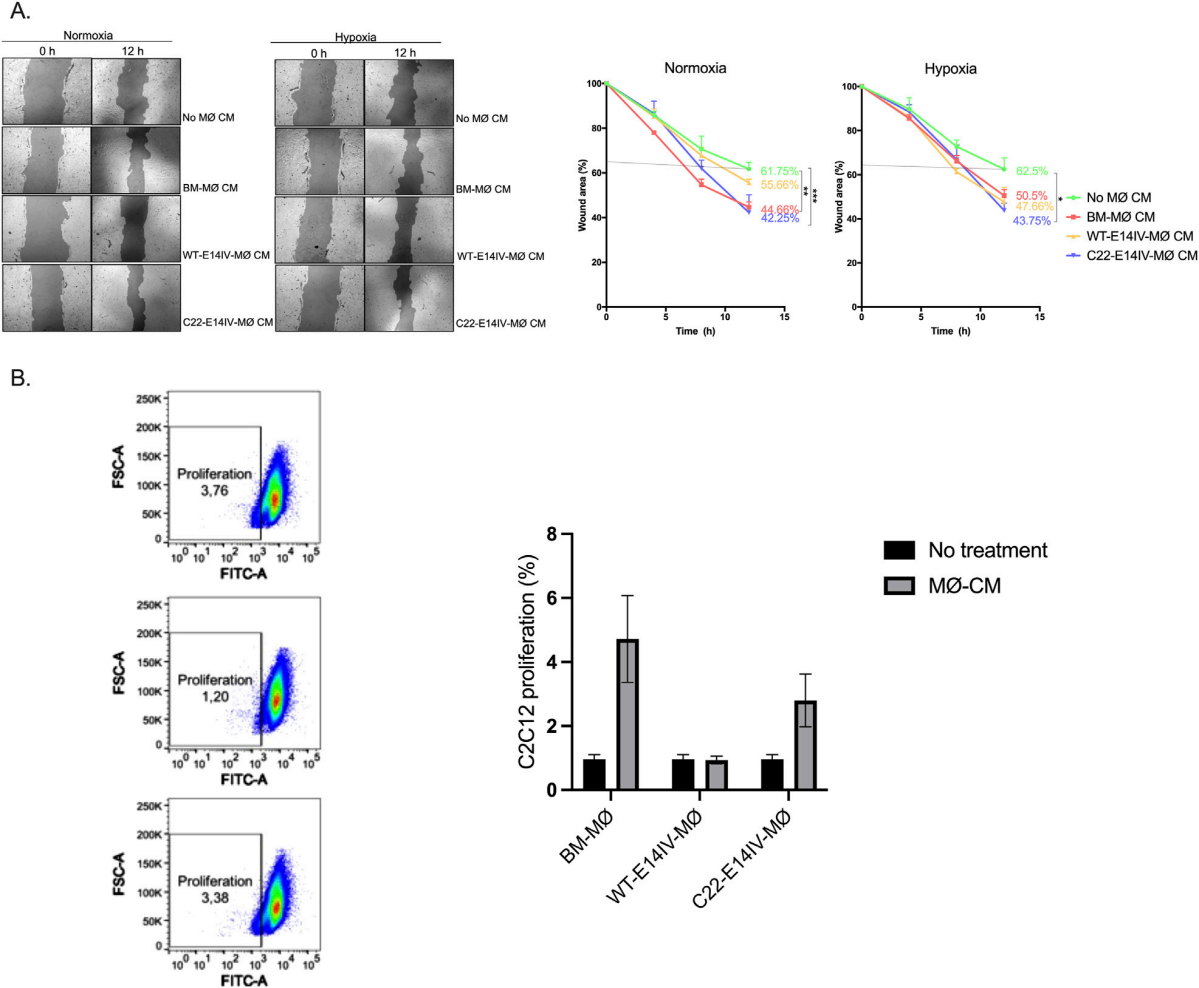
Paracrine effects of MØs on C2C12 cell migration and proliferation **(A)** Images from a wound-healing assay of C2C12 cells captured over a 12-h period following treatment with various MØ -conditioned media under normoxic and hypoxic conditions. The images were used to assess the wound closure rate. **(B)** Flow cytometry analysis of C2C12 cell proliferation after 24 h of treatment with MØ-conditioned media. Statistical analyses were performed by one-way ANOVA with Tukey’s multiple comparisons *post hoc* test (*p < 0.05; **p < 0.01; ***p < 0.001).

Considering that the proliferation and migration of myoblasts are essential for their differentiation into myotubes, which in turn contribute to tissue repair after ischemia, we investigated whether CM could stimulate these activities in the C2C12 myoblast cell line under normoxic and hypoxic conditions. The results from the scratch wound healing assay used to assess cell migration activity revealed that CM from both C22-E14IV-MØs and BM-MØs significantly increased migration under normoxic conditions compared with that in the control group (No MØ CM), with a statistically significant difference observed at the final time point (Figure 4A). Under hypoxic conditions, only the C22-E14IV-MØ-CM group showed a statistically significant difference compared with the control group; however, no significant differences were observed among the MØ groups. These findings suggest that CM from all MØ groups may have some ability to promote migration activity, particularly C22-E14IV-MØ-CM. However, as no statistically significant differences were observed among the MØ groups, further investigations are needed to confirm this effect.

To evaluate the effect of MØs on C2C12 cell proliferation, the cells were treated with CM from different MØ groups, and the cell numbers were assessed by flow cytometry after 24 h (Figure 4B). The cell proliferation rates remained below 5%, with no statistically significant differences observed among the groups, indicating that the CMs did not exert proliferative effects.

### 3.5 Therapeutic efficacy of MØ treatment for HLI

**FIGURE 5:**
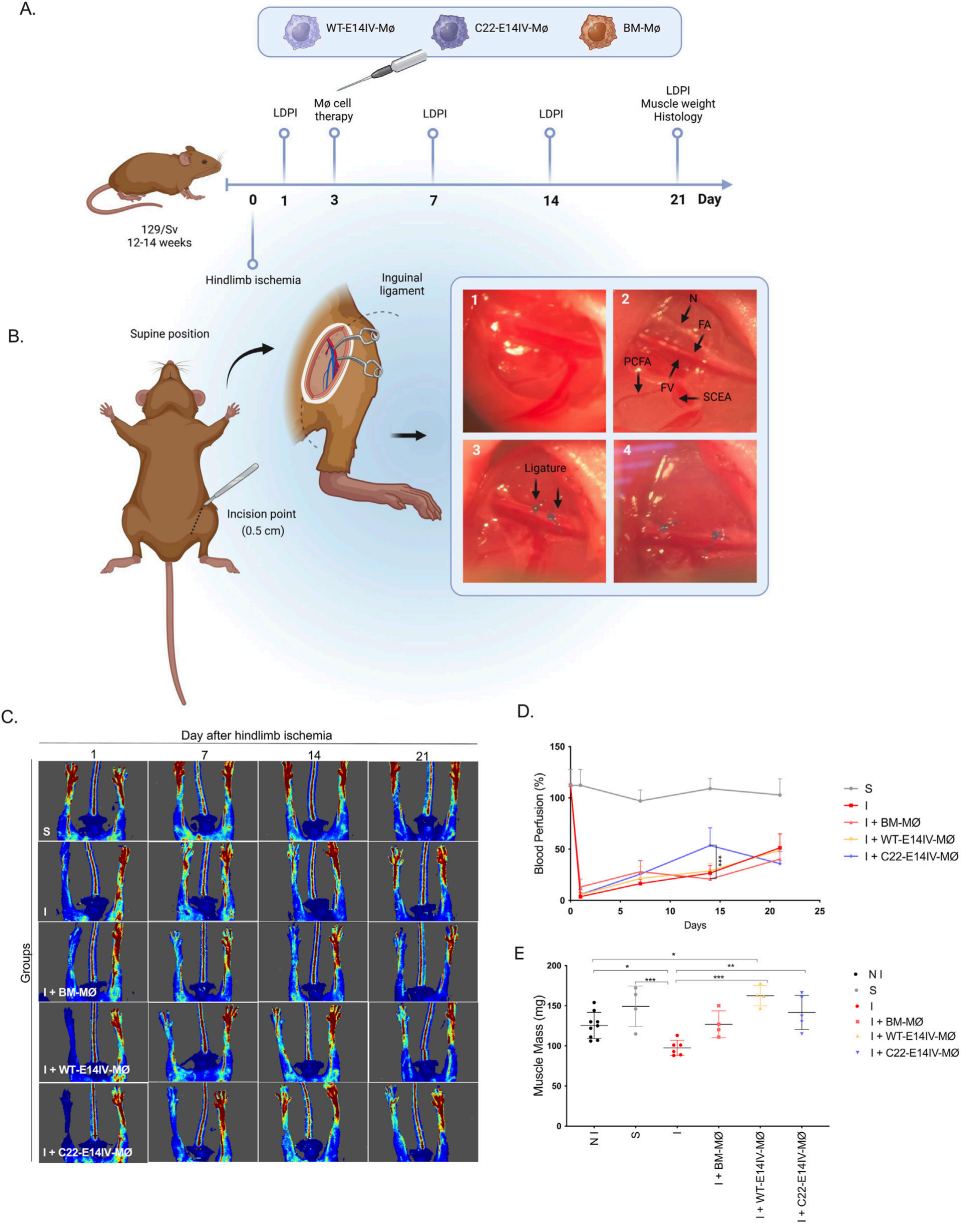
Functional effects of MØ therapy in a HLI model **(A)** Timeline of the *in vivo* experimental protocol. The presented steps were used to evaluate the effect of the proposed cell therapy in a HLI model. LDPI: Laser Doppler imaging. **(B)** Schematic representation of the mouse HLI surgery, indicating the site of the 0.5 cm incision at the level of the inguinal ligament. The images show the intact structure of the vessels (1); the structures of the femoral artery (FA), femoral vein (FV), nerve (N), proximal caudal femoral artery (PCFA), and superficial caudal epigastric artery (SCEA) (2); the site of the two ligatures on the FA (3); and the structures of the FA after transection between the ligatures (4). **(C)** Representative LDPI images of the groups: NI: nonischemic; S: sham-operated; I: ischemic; I + BM-MØ: ischemic treated with BM-MØs; I + WT-MØ-ES: ischemic treated with WT-E14IV-MØs; I + C22-MØ-ES: ischemic treated with C22-E14IV-MØs on Days 1, 7, 14, and 21. **(D)** Quantification of hindlimb perfusion on Days 1, 7, 14, and 21 after ischemia. **(E)** Gastrocnemius muscle mass assessment on the day of euthanasia. Data are expressed as mean ± SD. Blood perfusion over time **(D)** was analyzed using two-way ANOVA followed by Tukey’s *post hoc* test, while comparisons among groups in **(E)** were analyzed using one-way ANOVA followed by Tukey’s multiple comparison test. *p* < 0.05 was considered statistically significant (*p < 0.05; **p < 0.01; ***p < 0.001).

**FIGURE 6:**
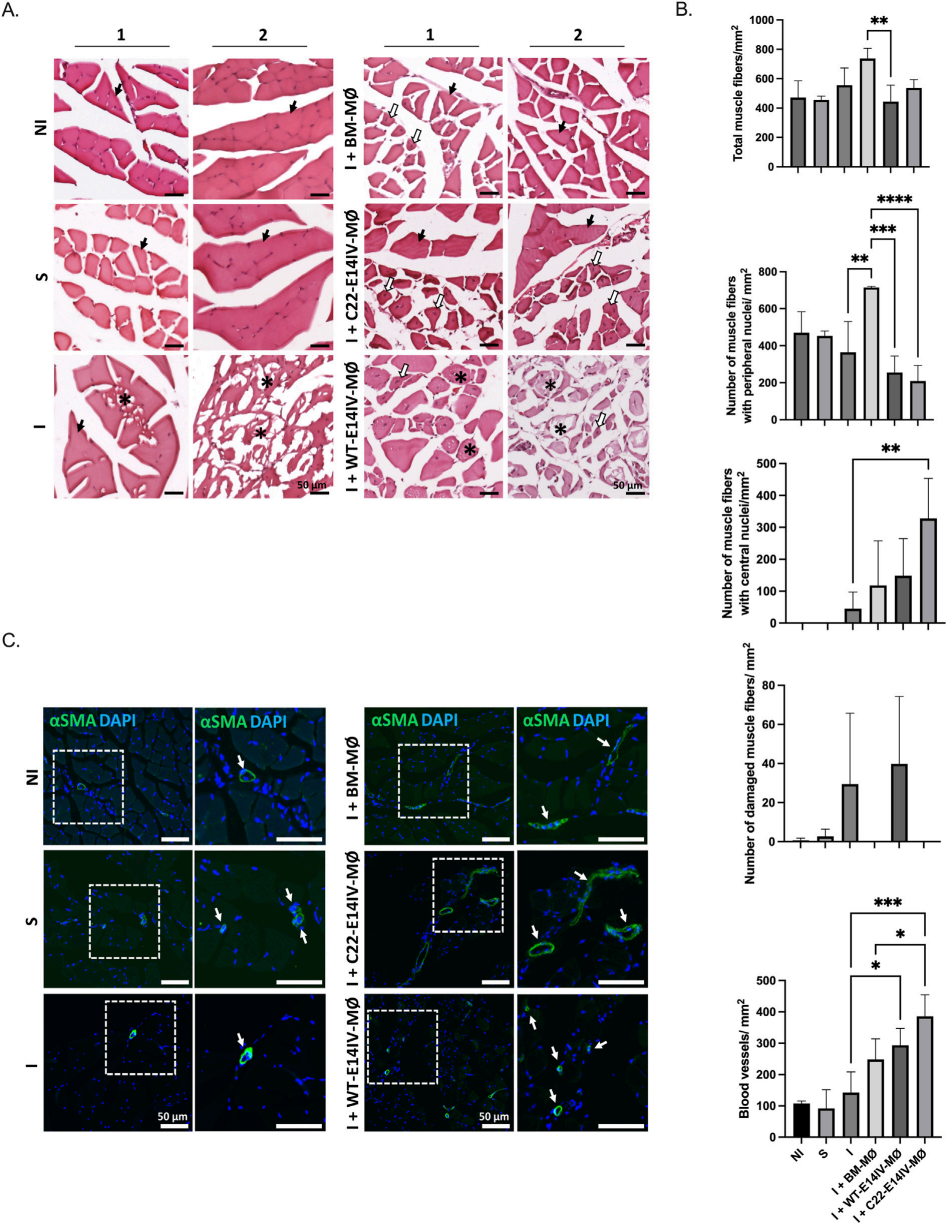
Histological analysis of the effects of MØ therapy for hindlimb ischemia Groups: NI: nonischemic; S: sham; I: ischemic; I + BM-MØ: ischemic treated with BM-MØs; I + WT-E14IV-MØ: ischemic treated with WT-E14IV-MØs; I + C22-E14IV: ischemic treated with C22-E14IV-MØs. **(A)** Representative images from two animals from each of the different experimental conditions. Normal-appearing polygonal skeletal striated muscle fibers and peripheral nuclei (black arrows); central nuclei (white arrows); degenerating muscle fibers (*); Staining: HE. **(B)** Data represent the means ± SDs of the total number of muscle fibers and the numbers of muscle fibers with peripheral nuclei and central nuclei. For blood vessel density, capillaries, venules, and arterioles were considered. (n = 3–5 animals/group). *p < 0.05, **p < 0.01 and ***p < 0.001 (ANOVA followed by the *post hoc* Bonferroni correction). **(C)** α-SMA-positive arterioles (green and arrows). Nuclei were stained with DAPI (blue).

To evaluate the therapeutic efficacy of MØs in a murine model of HLI, we injected the cells into the gastrocnemius muscle following surgical induction of ischemia. The timelines of all *in vivo* experiments and the details of the ischemia induction surgery are shown in Figures 5A,B, respectively. Blood perfusion was monitored weekly for 21 days (Figure 5). At the end of the study, muscle mass analysis (Figure 5) and histological analysis (Figure 6) were performed after euthanasia.

Laser Doppler images revealed that ischemia-inducing surgery drastically reduced blood perfusion immediately after the surgery. However, 1 week later, more than 10% of the blood perfusion had recovered, and this trend continued almost linearly, reaching a perfusion rate of approximately 45% after 3 weeks without any treatment (Figures 5C,D). Mice treated with BM-MØs and WT-E14IV-MØs followed a similar blood perfusion recovery profile, with no statistically significant differences between the groups over the 21-day period. However, on day 14, the C22-E14IV-MØ-treated group presented a statistically significant increase in blood perfusion compared with the other groups. By day 21, all the treated and untreated groups presented similar blood perfusion levels, with no significant differences among them.

Loss of muscle mass caused by ischemia was evaluated by weighing muscles obtained immediately after euthanasia. Compared with that in the sham-operated and nonischemic groups, the muscle mass in the ischemic group was significantly lower (NI: 125 ± 16; S: 149 ± 25; I: 97.5 ± 9). Notably, treatment with C22-E14IV-MØs and WT-E14IV-MØs significantly improved muscle mass recovery compared with that in the untreated ischemic group (I + BM-MØs: 127 ± 17; I + C22-E14IV-MØs: 142 ± 21; I + WT-E14IV-MØs: 163 ± 13), with values similar to those of the sham-operated group (Figure 5E).

To evaluate muscle recovery status, muscles collected post-euthanasia were subjected to histological analysis. This analysis involved assessing the location and number of nuclei within each muscle fiber, the extent of fibrosis, and the quantity and size of vessels. These parameters were evaluated using images of samples after HE and Masson’s trichrome staining (Figures 6A,B), along with staining with an *α*-SMA antibody (Figure 6C). Histomorphometry revealed that treatment with BM-MØs increased the total number of muscle fibers, although the difference was not statistically significant in relation to the control groups (NI and I) and C22-E14IV-MØs. Among the observed fibers, mature fibers with peripheral nuclei constituted the primary fiber population in the BM-MØ group, indicating that muscle regeneration was much less active. On the other hand, the group treated with C22-E14IV-MØs presented fewer fibers with peripheral nuclei and more fibers with central nuclei than the I-group did. This finding revealed a greater number of active fibers and fewer mature fibers, indicating that these muscles are still undergoing regeneration. Damaged muscle fibers were present in the ischemic and WT-E14IV-MØ groups, but these fibers represented a very small proportion of total fibers, and the obtained values were not significantly different from those in the other groups, indicating that the presence of damaged fibers was negligible after 3 weeks of ischemia, regardless of the treatment. In relation to the control groups, there were no significant differences between the NI and Sham groups, as expected. The total number of muscle fibers was similar between the I group and the NI and S groups, revealing a full recovery in terms of the total number of fibers; these fibers were composed mainly of mature fibers. Notably, none of the evaluated groups exhibited regions of fibrosis.

Angiogenesis and arteriogenesis, which are essential for blood flow, are critical factors in muscle recovery following ischemia. The number of blood vessels increased after MØ therapy, and this effect was further augmented by C22-E14IV-MØ treatment (Figure 6B). When counting blood vessels, all capillaries, venules, and arterioles were considered; however, capillaries predominated in terms of number. Notably, the vessels in the C22-E14IV-MØ-treated group were significantly larger than those in the other groups (Figure 6C), indicating more arteriogenesis.

## 4 Discussion

Our study highlights the successful generation of Phd2-haplodeficient MØs that exert proresolution effects in the treatment of ischemic muscles. To achieve this goal, we knocked out one allele of the *Egln1/Phd*2 gene in the E14IV-ES ESC line using CRISPR‒Cas9 technology, generating ESCs that differentiated into MØs that expressed a reduced level of Phd2. Given that testing therapeutic efficacy in large animals, including humans, necessitates a large quantity of MØs, we initiated this project with an ESC line because it can be expanded indefinitely before differentiation into MØs (Haideri et al., 2017). Furthermore, since ESCs exhibit characteristics similar to those of iPSCs (Yamanaka, 2020), the latter can be generated from a patient and utilized for their own treatment to reduce immune reactions. Regardless of the stem cell source for producing MØs, ensuring that pluripotent cells are absent in the resultant MØs is crucial because of biosafety concerns. In our study, we utilized the ESC marker SSEA-1 (Cui et al., 2004) to verify this, and no pluripotent cells were detected in the C22-E14IV-MØ samples (Figure 2A).

Under normoxic conditions, haplodeficient C22-E14IV-ES cells and C22-E14IV-MØs expressed Phd2 at a level of approximately 50% relative to that of their parental E14IV-ES cells and their derivative WT-E14IV-MØs, indicating stoichiometric changes in gene expression, as expected. However, under hypoxic conditions, Phd2 protein expression remained at a level similar to that under normoxic conditions, suggesting that the Phd2 expression system in haplodeficient cells operates as it does under hypoxic conditions, independent of oxygen tension variation. The Phd2 expression profile of haplodeficient MØs indicates that we achieved the purpose of this study, which was to generate MØs that express Phd2 under normoxic conditions while mimicking a hypoxic state.

The consistent ∼50% reduction in Phd2 protein levels in edited cells confirms that the CRISPR/Cas9-generated allele behaves as a functional *Egln1* haplodeficient allele. Because the edited segment lies within a non-coding intronic region, far from canonical splice donor or acceptor motifs, a direct effect on splicing is unlikely. Intronic indels have been reported to subtly influence gene expression through mechanisms involving local chromatin structure, transcriptional efficiency, or non-coding regulatory elements. Although the present data do not allow us to define the exact mechanism, the reduction in Phd2 protein levels is consistent with a mild cis-acting effect on Egln1 expression. Previous studies have shown that non-coding regions of *EGLN1*, including proximal intronic elements, can modulate its transcriptional output under specific physiological contexts (Aggarwal et al., 2010; Heinrich et al., 2019; Xin et al., 2020). Our observations align with the concept that non-coding segments of the locus may contribute to quantitative regulation of Egln1 expression, which is compatible with the generation of a stable haplodeficient state.

The C22-E14IV-MØs generated under our experimental conditions predominantly exhibited an M2-like phenotype, as they were positive for *Arg1*, *Fizz*, and CD206 but lacked expression of M1-associated genes, including *iNos*, *Cd86*, and *Stat1*. However, approximately half of this population also expressed MHCII, a marker typically associated with M1 MØs (Mills, 2015), indicating the presence of a mixed population with an ambiguous identity. However, recent research has demonstrated that MØs with high MHCII expression can be polarized into diverse subtypes that modulate inflammation on the basis of the surrounding physiological and pathophysiological conditions (Cole et al., 2018; Shapouri-Moghaddam et al., 2018; Wei et al., 2023; Weinberger et al., 2021). In particular, studies on bronchoalveolar lavage fluid from asthma patients have revealed a significant increase in M2 MØs coexpressing CD206 and MHCII (Girodet et al., 2016). This finding reinforces the complexity of MØ polarization and suggests that the C22-E14IV-MØ population is not exclusively M2 but comprises at least two main subsets with seemingly opposing identities on the basis of MHC class II expression. However, further studies are needed to clarify the functional roles of these cells in both physiological and pathological contexts. This finding also highlights that categorizing MØ populations as only M1 or M2 is inadequate, and such classifications should be contextualized within the physiological and pathophysiological context.

Phagocytosis is a key MØ function, particularly in resolving inflammation, which in turn supports muscle regeneration. One of the mechanisms involved in this process is phagocytosis-mediated MØ modulation, shifting from a proinflammatory to an anti-inflammatory profile, which is mediated by AMPKα1 during the resolution of inflammation (Mounier et al., 2013). According to Haideri et al. (Haideri et al., 2017), ESC-derived MØs exhibit weaker phagocytic activity than do bone marrow-derived MØs, but here, we showed that a reduction in Phd2 expression in ESC-derived MØs resulted in an increase in phagocytic activity, suggesting that Phd2 haplodeficient MØs may adopt a more anti-inflammatory and proresolution phenotype.

Angiogenesis is one of the key processes required to resolve ischemia. C22-E14IV-MØs exhibited a notable ability to secrete a range of angiogenic factors (Figure 3A), which likely contributed to the stimulation of endothelial cell tube formation (Figure 3B). However, the CM from C22-E14IV-MØs did not promote C2C12 cell proliferation, and its effect on migration was only significant compared with that of the control group (No MØ-CM), with no significant differences observed among the MØ groups (Figure 4). Previous studies have shown that coinjection of MØs with myoblasts enhances the survival, expansion, and migration of transplanted cells in dystrophic muscle (Lesault et al., 2012). However, proinflammatory MØs, in particular, have been reported to increase the regenerative capacity of myoblasts by extending their proliferation window, increasing their migration, and delaying their differentiation (Bencze et al., 2012). The role of M1-like MØs in muscle injury resolution was also observed in a traumatic muscle injury model after transfection with a vector expressing GM-CSF, a factor known to promote proinflammatory MØs (Martins et al., 2020). Given that C22-E14IV-MØs presented more M2-like markers, likely indicating a predominantly anti-inflammatory phenotype, the weak promotion of C2C12 cell proliferation and migration observed here appears to be consistent with the findings of previous studies. It is important to note that the interaction between macrophages and C2C12 myoblasts in this study was evaluated using conditioned media, a well-established approach that allows assessment of soluble paracrine factors while minimizing confounding cell–cell effects. Although Transwell co-culture systems could provide additional insight into bidirectional communication, our method already captures the main paracrine component of macrophage–myoblast crosstalk. Future studies employing Transwell or 3D co-culture systems could further elucidate the reciprocal and time-dependent aspects of macrophage–myoblast communication during muscle regeneration.

To assess the extent to which these *in vitro* effects could be translated to an *in vivo* limb ischemia model, we used 129SV/WT mice (Nagy et al., 1993), which share the same origin as E14IV-ES cells and consequently C22-E14IV-MØs, thereby minimizing host immune reactions. When evaluated in an ischemic muscle model, C22-E14IV-MØ therapy significantly improved ischemia recovery in comparison to that observed after all other treatments. This finding was confirmed by enhanced blood perfusion recovery and increased numbers of arterioles, which are characteristic of arteriogenesis (Hamm et al., 2013; Takeda et al., 2011). In this study, we utilized a mild limb ischemia model (Gallo et al., 2023) to prevent limb loss; therefore, circulation in untreated ischemic limbs is expected to recover approximately 3 weeks after ischemic surgery, as was observed in this study (Figures 5C,D). However, a notably faster recovery of blood circulation was observed in the second week post-therapy with *Phd2* ^+/−^ MØs, indicating a substantial improvement in ischemic muscle conditions, as confirmed by histological analysis. This improvement can likely be attributed, at least in part, to the presence of a significantly greater number of active muscle fibers than in the other groups, suggesting that an ongoing regenerative process could further enhance muscle recovery.

Overall, this study highlights the successful generation of Phd2-haplodeficient MØs with proresolution effects for the treatment of ischemic diseases. The distinct characteristics exhibited by C22-E14IV-MØs, such as increased phagocytosis, secretion of angiogenic factors, and promotion of endothelial cell tube formation, provide mechanistic insights into their therapeutic effects. The observed improvements in *in vivo* ischemia recovery, such as increased blood perfusion and vessel formation, further emphasize the potential clinical relevance of *Phd2* ^+/−^MØ therapy. Further investigations exploring the long-term effects and safety of these cells are crucial for their translation to clinical applications. Additionally, these findings provide new perspectives for the use of iPSCs as a source of patient-specific *Phd2* ^+/−^ MØs, offering the dual advantages of reducing immune responses to these cells and enabling large-scale production.

## Data Availability

The original contributions presented in the study are included in the article/Supplementary Material, further inquiries can be directed to the corresponding author.
