# Supplementary material for: Generation of Phd2-haplodeficient macrophages with proresolution effects for the treatment of limb ischemia
Source: Front Pharmacol. 2025 Dec 19;16:1698623. doi: 10.3389/fphar.2025.1698623 (PMC12757696; doi:10.3389/fphar.2025.1698623)

**SUPPLEMENTARY DATA**

**Table S1. Information on gRNA design**

| Names | Strands | Sequences | Off-targets |
| --- | --- | --- | --- |
| PHD2-gRNA1 | 1 | ATGAGCCAAC ATGGAAGGTC AGG | 0: 1, 1: 0, 2: 0, 3: 9, 4: 164 |
| PHD2-gRNA2 | 1 | ATGGAATGAG CCAACATGGA AGG | 0: 1, 1: 0, 2: 0, 3: 12, 4: 240 |
| PHD2-gRNA3 | -1 | CTACGCCGTG TTTTTTATAA TGG | 0: 1, 1: 0, 2: 0, 3: 1, 4: 38 |

**Table S2. Primers used for RT-qPCR**

| Gene  name | Gene Data  Bank | Forward | Reverse | Efficiency | |
| --- | --- | --- | --- | --- | --- |
| *Pu.1* | AB117644.1 | CAGACACCAATGGACACTCG | GGGCTGGTTTTCAACAAGG | | 1,99 |
| *Stat1* | BC004808 | GAACGGAAGCATTTGGAATC | CATGGGGAAACTGTCATCG | | 1,94 |
| *Stat6* | BC127054.1 | CTGCGAACCCTTGTGACC | TTGGCTGAGGTCCCTAGAAA | | 1,92 |
| *iNos* | BC053416 | AACACCCGGGACATGAGAC | CCTGGGAGGATCAGGAAGTC | | 2,1 |
| *Cd86* | BC013807.1 | GAAGCCGAATCAGCCTAGC | CAGCGTTACTATCCCGCTCT | | 2,0 |
| *Arg1* | BC013341.1 | GAATCTGCATGGGCAACC | GAATCCTGGTACATCTGGGAAC | | 1,99 |
| *Fizz* | AF316397.2 | TATGAACAGATGGGCCTCCT | AGGCAGTTGCAAGTATCTCCA | | 1,98 |
| *Gapdh* | BC023196.2 | CATCTTCTTGTGCAGTGCCA | CGGCCAAA TCCGTTCAC | | 2,02 |

PCR efficiency was calculated using the LightCycler 480 software

**Fig. S1. Quantification of Phd2 protein expression on clones from E14IV-ES *Phd2^+/-^***

Protein expression was quantified relative to the WT, which was assumed to express 100% of the Phd2 protein.


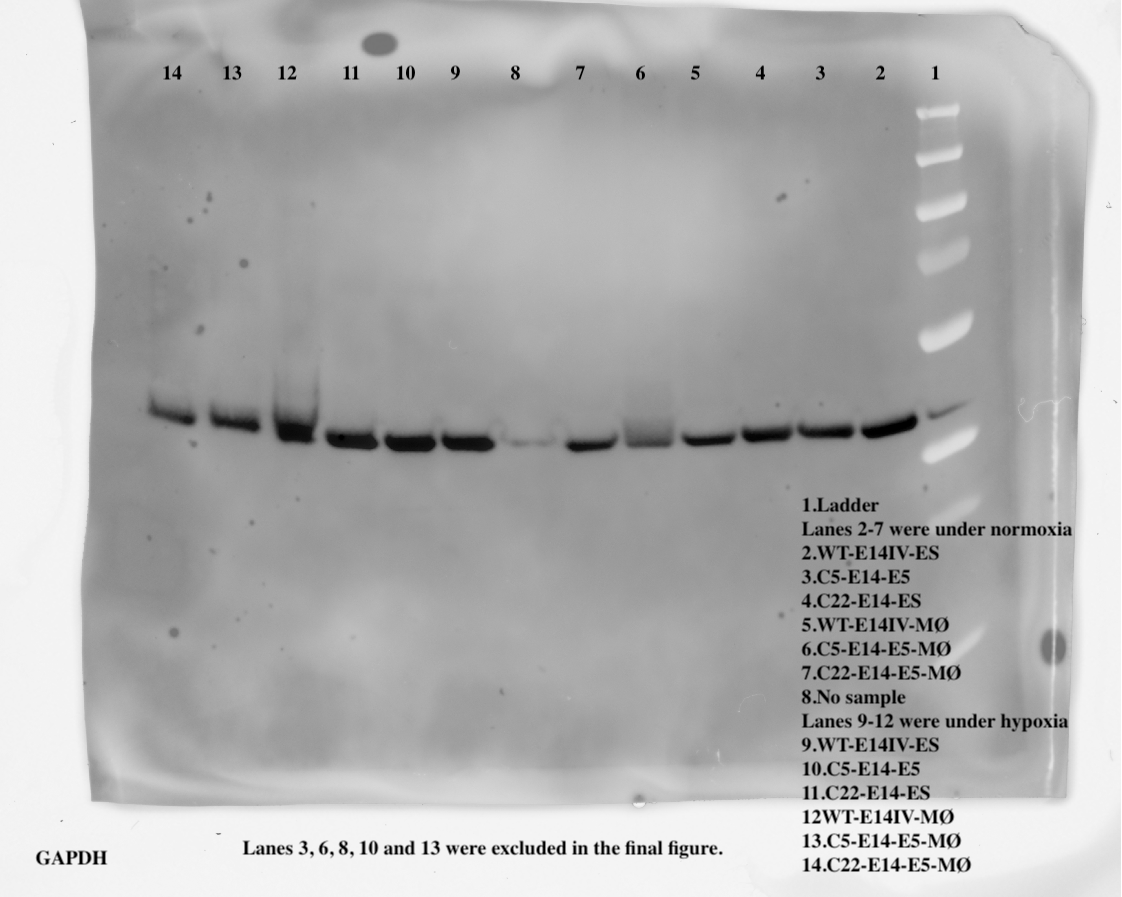


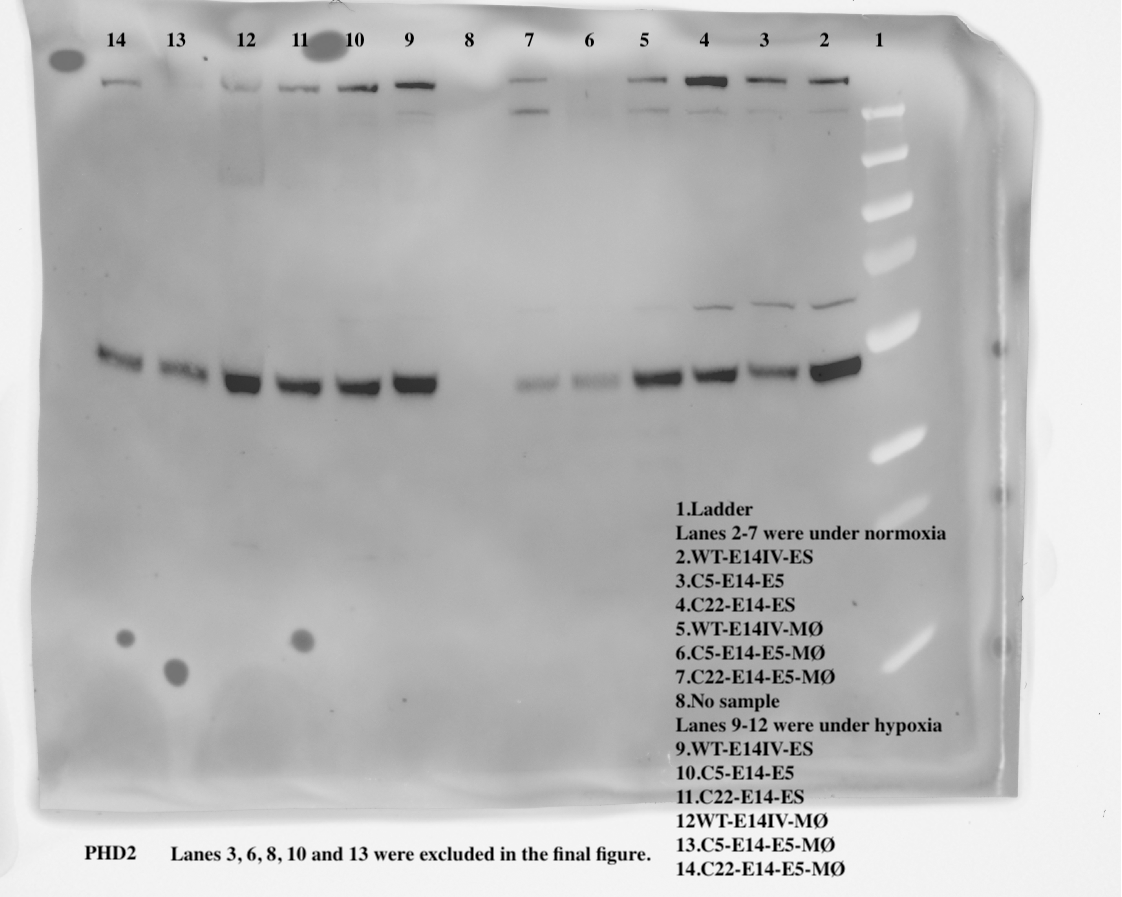


**Fig. S2.** Enlarged representative images of undifferentiated E14IV-ES cells and differentiated macrophages shown in Figure 1
Phase-contrast microscopy images illustrating the typical morphology of undifferentiated E14IV-ES cells and macrophages differentiated for 14 days. Scale bars: 100 µm.


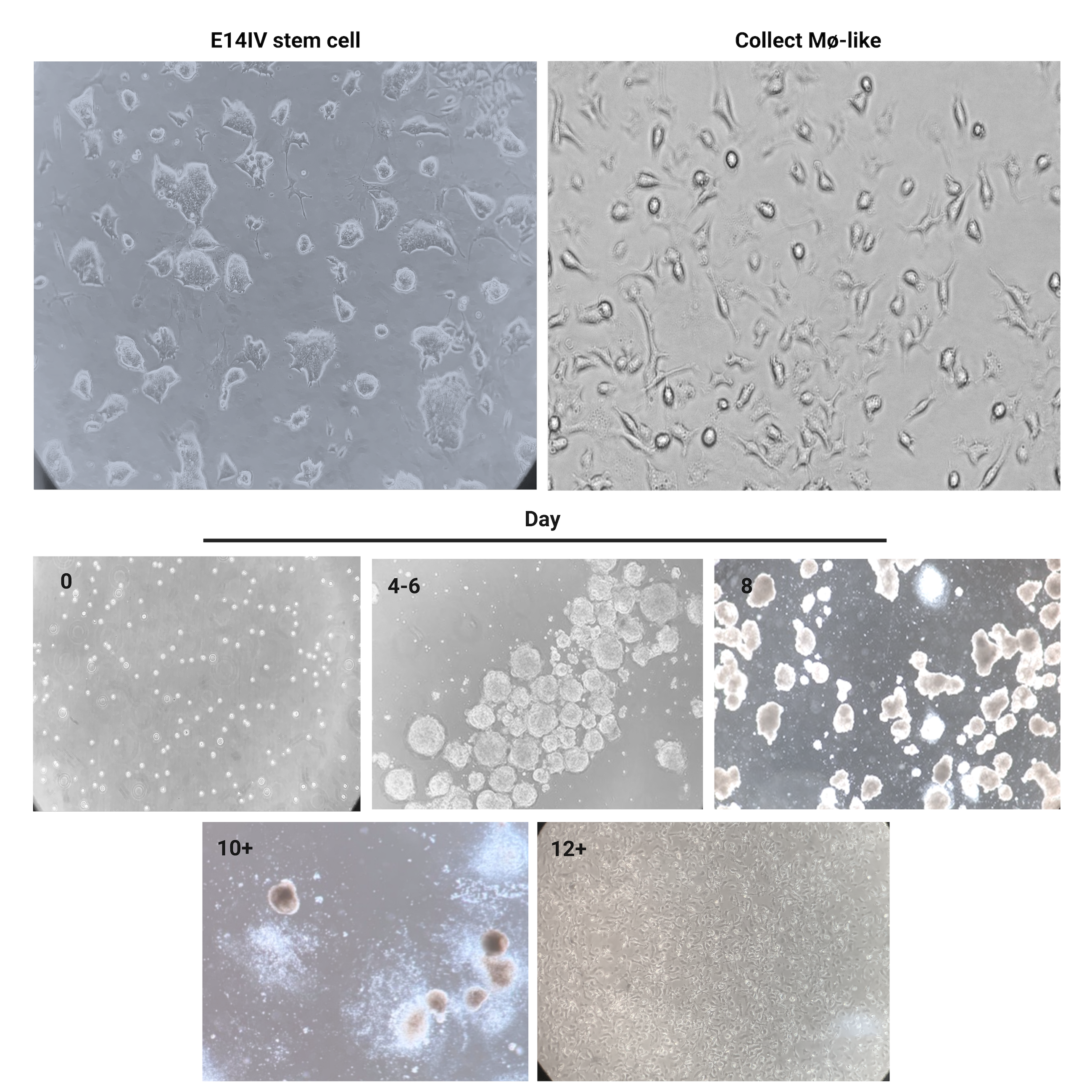


**Fig. S3.** Time-lapse imaging of phagocytosis by WT-E14IV-MØs and C22-E14IV-MØs
Representative live-cell fluorescence microscopy images showing the time-dependent uptake of pHrodo™ Green Zymosan bioparticles by WT-E14IV-MØs and C22-E14IV-MØs. Images were captured at 0, 20, 40, 60, 80, and 100 minutes after bead addition.


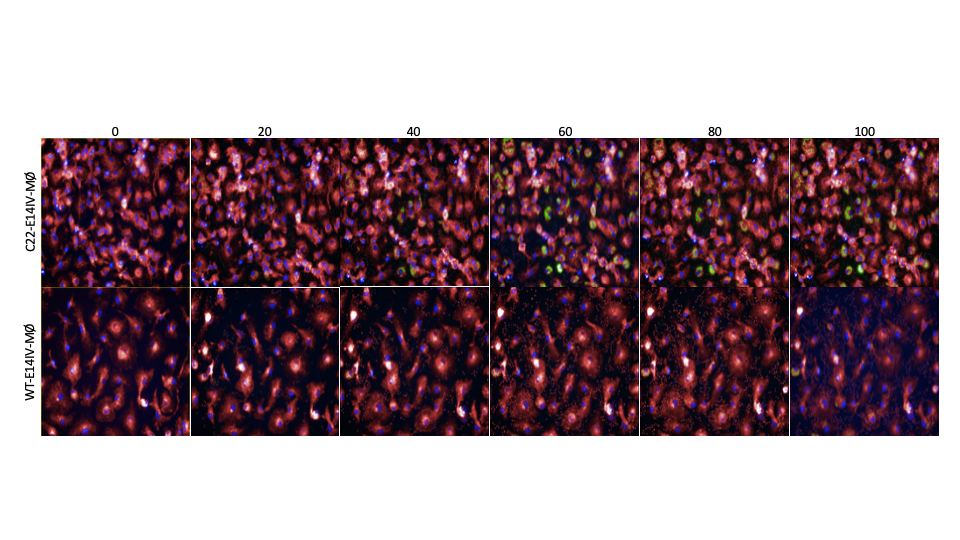

Supplement: Supplementary file 1 [file Supplementaryfile1.docx]
